# Pore Architectures and Mechanical Properties of Porous α-SiAlON Ceramics Fabricated via Unidirectional Freeze Casting Based on Camphene-Templating

**DOI:** 10.3390/ma12050687

**Published:** 2019-02-26

**Authors:** Zhaoping Hou, Feng Ye, Qiang Liu, Limeng Liu, Haiwei Jiang, Shaowei Zhang

**Affiliations:** 1 College of Materials Science and Engineering, Taiyuan University of Technology, Taiyuan 030024, China; jianghaiwei@tyut.edu.cn; 2 School of Materials Science and Engineering, Harbin Institute of Technology, Harbin 150001, China; yf306@hit.edu.cn (F.Y.); qiangliu@hit.edu.cn (Q.L.); 3 School of Materials Science and Engineering, North Minzu University, Yinchuan 750021, China; llm681@sina.com; 4 College of Engineering, Mathematics and Physical Sciences, University of Exeter, Exeter EX4 4QF, UK; S.Zhang@exeter.ac.uk

**Keywords:** camphene-templated porous α-SiAlON, porosity, pore size, mechanical properties

## Abstract

Porous α-SiAlON ceramics were fabricated using the camphene-based unidirectional freeze casting method, in which a gradient porous structure was formed as a result of the decreased solidification velocity in the freezing direction. Microstructure, porosity and pore size distribution of different parts of as-prepared samples were examined and compared, and correlated with their mechanical properties. For a given solid loading, the overall pore size and porosity of the top part were greater than those of the bottom part. Interestingly, despite its higher porosity, the flexural strength and fracture toughness of the top part were both higher than those of the bottom part, suggesting that apart from porosity, pore morphology and size affected mechanical properties of as-prepared porous α-SiAlON ceramics.

## 1. Introduction

Freeze casting has attracted a great deal of attention, as a porous ceramic processing technique, since it can create various porous structures via controlling/tuning processing parameters, such as solvent type, solid loading, suspension viscosity, and freezing rate [1,2]. In this technique, a ceramic slurry is usually solidified under a unidirectional temperature gradient, and the subsequent freeze-drying results in an anisotropic porous microstructure which can be retained to a large extent after densification via conventional sintering routes. A significant number of studies focused on the inherent process-microstructure correlations. The relationship between solidification velocity and pore microstructure was reported for different freezing vehicles like water, camphene and tert-butyl alcohol (TBA) [1,2,3,4]. For camphene-based slurry, the increase in solidification velocity causes a significant decrease in the sizes of dendrite pores and the secondary arm dendrite spacing (SDAS) [5].

For porous ceramics prepared by freeze casting, understanding their structure-property (mechanical properties, in particular) relationships is also fundamentally and technically important. For ice-templated scaffolds, their mechanical performance is affected by a number of factors such as porosity, pore size, aspect ratio and morphology, lamella walls roughness and interlamellae bridge density [6,7,8,9]. The strength increases with the decrease of porosity. In addition, the strength can be enhanced by increasing the interlammellae bridge density, and decreasing the pore size [8,9]. However, little work has been carried out on the relationship between the microstructure and mechanical properties of sintered camphene-templated scaffolds.

In this paper, porous α-SiAlON ceramics were prepared via unidirectional freezing of camphene-based suspensions. Owing to the thermal resistance of the solidified layer, the solidification velocity decreased during the freezing process, resulting in different pore sizes and pore morphologies at different heights of the sample [10,11], which were examined and compared, and further correlated with mechanical properties of the samples.

## 2. Experimental

### 2.1. Preparation of Ceramic Slurries 

α-Si_3_N_4_ (containing 1.27% oxygen, SN-E10, UBE Industries, Yamaguchi, Japan), Al_2_O_3_ (>99.9%, 0.5 μm, A16-SG, Alcoa, Massena, NY, USA), AlN (grade C, H.C. Stark, Berlin, Germany), and Y_2_O_3_ (>99.9%, grade fine, H.C. Stark, Berlin, Germany) were used as the main starting materials to fabricate porous α-SiAlON (Y_m/3_Si_12−(m+n)_Al_m+n_O_n_N_16−n_, m = 1.0, n = 1.0 ) ceramics. 

For ceramics slurries (30 vol% solids content), requisite amounts of the raw material powders for forming the target SiAlON, and 1 wt.% (with respect to the raw material powders) HAO FAST 923 dispersant (Shanghai Haoyang Co., Ltd., Shanghai, China) were mixed with camphene solvent (95% Purity, Guangzhou Huanpu chemical Factory, Guangzhou, China), followed by ball milling at 60 °C for 24 h in a sealed plastic bottle. 

### 2.2. Freeze Casting and Firing

Unidirectional freeze casting was performed by casting the resultant warm slurries into polyethylene molds (60 mm in diameter and 20 mm in height) placed on a copper plate, immersed in ice water at 0 °C. The polyethylene tubes (molds) were radially insulated and pre-warmed at 60 °C to avoid horizontal (radial) thermal gradient and ensure that the camphene crystals grew only from the cold bottom and under the vertical (unidirectional) temperature gradient.

After freeze casting, the frozen samples were removed from the molds and placed on a polyurethane sponge in open air at room temperature for about four days to sublime the solidified camphene in the green compacts, before being fired in a graphite resistance furnace at 1900 °C for 1 h in N_2_ with a pressure of 0.6 MPa. The heating and cooling rates were both 10 °C /min.

### 2.3. Microstructural Characterization, Porosity Examination, and Mechanical Testing

**Figure 1:**
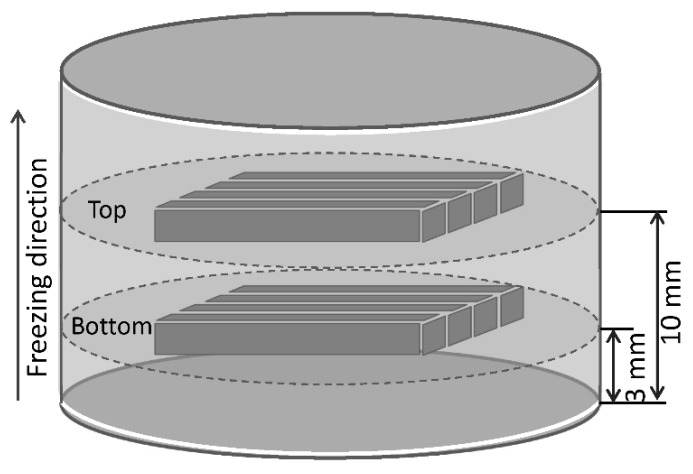
Schematic illustration of the locations from where the mechanical testing specimens were taken.

Microstructures of different parts of each fired porous sample (at the distances of 0.1, 3, and 10 mm, from the bottom of each sample) (Figure 1) were examined by a scanning electronic microscope (SEM, Quanta 200FEG, FEI, Hillsboro, OR, USA). Phases in fired samples were identified by X-ray diffraction (XRD, RTP 300, Rigaku, Tokyo, Japan) analysis. Flexural strength and fracture toughness were determined by three-point bending and a single-edge-notched beam (SENB) test (Instron-5569, Instron, Norwood, MA, USA), respectively. For each fired porous α-SiAlON sample subjected to mechanical testing, four bar specimens were extracted from two different sections (referred to as bottom and top parts) transverse to the freezing direction, as shown in Figure 1. The dimensions of specimens for the flexural strength and fracture toughness tests were 3 mm × 4 mm × 36 mm and 2 mm × 4 mm × 20 mm, respectively. The open porosity and pore size distribution of the bar specimens were measured using a mercury porosimetry (Autopore9500, Micromeritics Co., Norcross, GA, USA)

## 3. Results and Discussion 

### 3.1. Phase Formation

**Figure 2:**
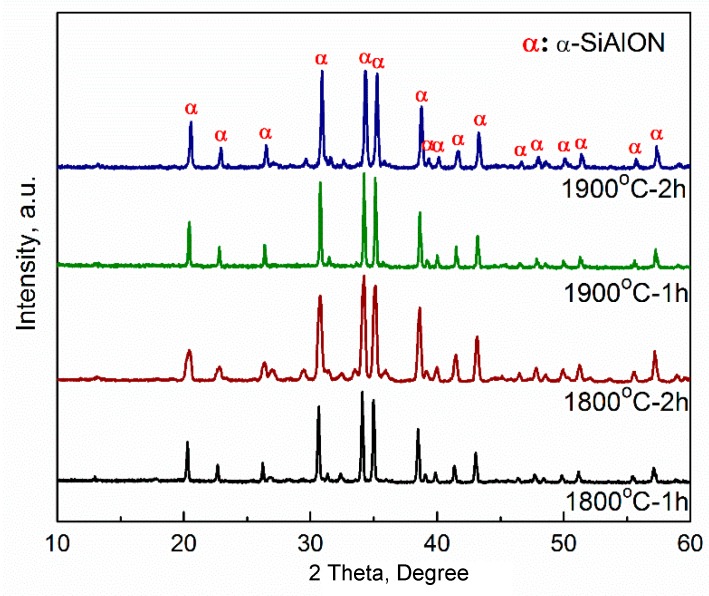
XRD patterns of porous α-SiAlON samples fired at different conditions.

Figure 2 shows XRD results of porous α-SiAlON samples fired at different conditions, revealing very similar patterns and the formation of nearly phase pure α-SiAlON in each sample, which further indicated essentially the completion of the α-SiAlON formation reaction between Si_3_N_4_, Al_2_O_3_, Y_2_O_3_ and AlN. In addition, it is noted that that the α-phase peaks of porous α-SiAlON sample fired at 1800 °C for 2 h were broader than those of other samples. Unfortunately, the responsible mechanism for this is still not clear, which warrants further investigation by the future work.

### 3.2. Microstructure and Porosity

**Figure 3:**
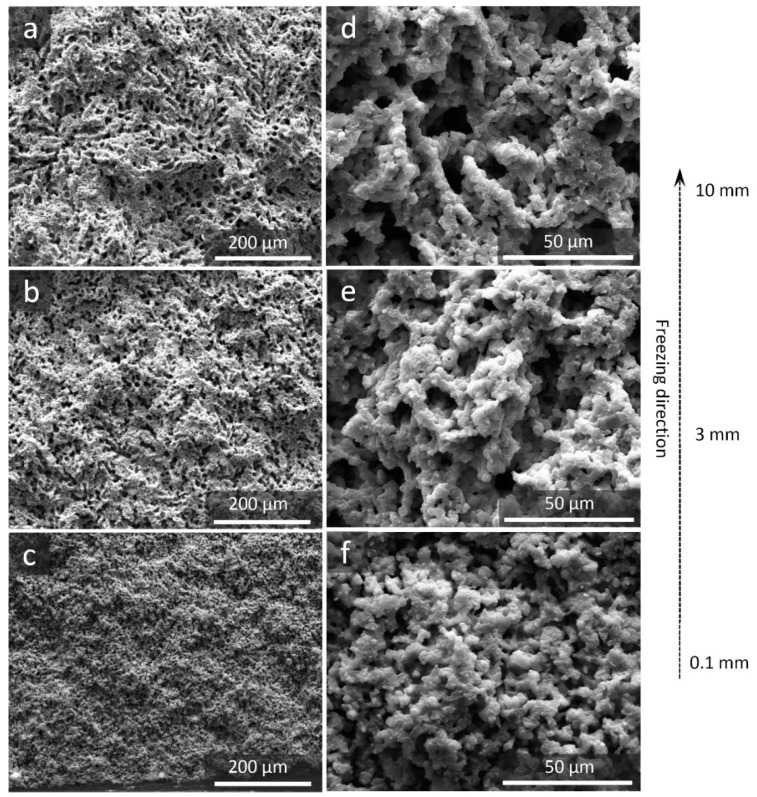
SEM images of porous α-SiAlON samples fired at 1800 °C for 1 h. (**c**,**f**), (**b**,**e**) and (**a**,**d**) are respectively the longitudinal section at approximately 0.1, 3 and 10 mm from the bottom.

Figure 3 shows SEM images of a longitudinal section of the porous α-SiAlON sample fired at 1800 °C for 1 h. A structural gradient was evident where the pore morphology and size varied as a function of the distance from the bottom of the sample. At the distance of 0.1 mm where the solidification initiated, rapid and homogeneous nucleation of solvent crystals occurred, resulting in an irregular pore structure. At the distance of 3 mm, the primary crystals grew with side-branching or secondary dendritic growth, giving rise to a dendritic pore morphology. Furthermore, the pore size increased significantly with increasing the distance from 3 to 10 mm. The change of microstructural feature in the freezing direction is typical in the case of a camphene-templated scaffold and attributable to the difficulty in maintaining a constant solidification velocity over long distance (cm) under the unidirectional freezing condition [11]. Due to the thermal resistance of the solidified layer, the solidification velocity decreased with increasing its thickness, resulting in increased pore size and the secondary dendrite arm spacing (SDAS). 

**Figure 4:**
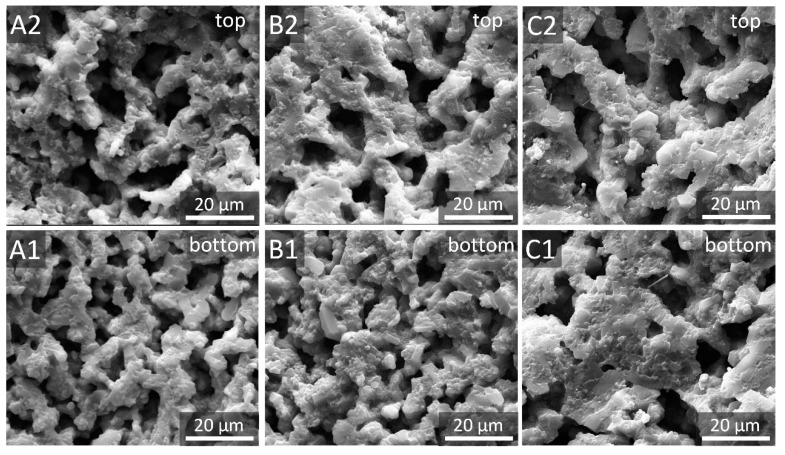
SEM images of the top and bottom parts of porous α-SiAlON samples fired under different conditions: 1800 °C-2 h (**A1**, **A2**), 1900 °C-1 h (**B1**, **B2**) and 1900 °C-2 h (**C1**, **C2**).

Figure 4 compares microstructures of the top (at the distance of 3 mm) and bottom parts (at the distance of 10 mm) of porous α-SiAlON samples fired under different conditions. Irrespective of the firing condition, dendrite pores were formed in the top part, and their overall size was bigger than that of the bottom part. With increasing the firing temperature and time, the pore walls tended to become denser and their thickness tended to increase. Moreover, the pore size in the top part changed very little even after 2 h firing at 1900 °C (Figure 4C2), but the smaller and more intricate pores in the bottom part almost disappeared and were replaced by much bigger pores (Figure 4C1). This could be attributed to the fact that the smaller and more intricate pores had a greater driving force for densification because of the smaller radius of curvature of the interface [12].

**Figure 5:**
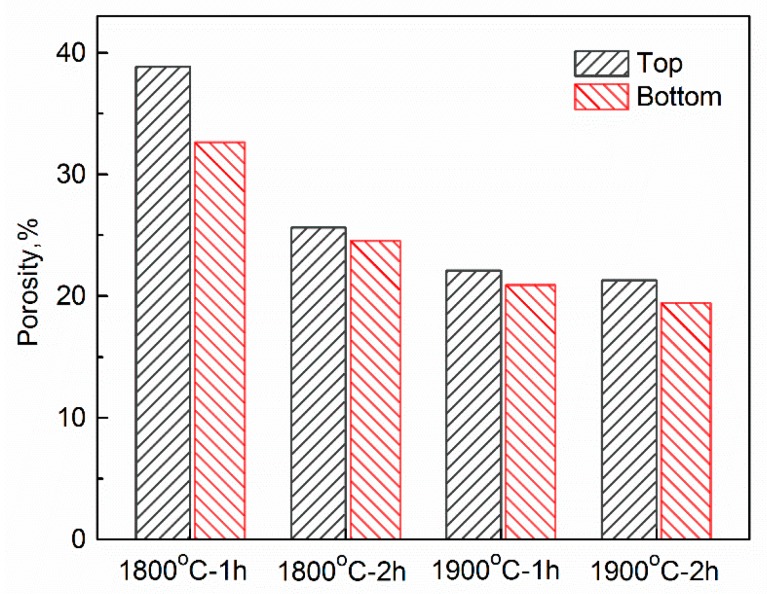
Porosities of the top and bottom parts of porous α-SiAlON samples fired at different conditions.

Figure 5 presents porosities of different parts of porous α-SiAlON samples fired under different conditions. As expected, with increasing the firing temperature and time, porosities of both (top and bottom) parts decreased. Furthermore, for each sample, the porosity in the top part was higher than in the bottom part. This could be explained by the particle redistribution and concentration behavior during the freezing process. 

With unidirectional freezing of ceramic slurries, camphene crystals form along the solidification direction, pushing the ceramic particles between the two dendrite arms to form ceramic walls until the particle redistribution ceased, where the volume of fraction particle reaches a critical value *φ_b_* (referred to as breakthrough concentration). After the sublimation step, the breakthrough concentration therefore defines the balance between the relative proportion of macropores, corresponding to camphene crystals, and micropores, corresponding to the interparticle spaces in the walls [12]. During the sintering process, the micropores are readily removable. Therefore, the porosity of a fired porous sample decreased with increasing the fraction of micropores. According to Deville et al. [5,12], the breakthrough concertation *φ_b_* can be expressed as

(1) $$\varphi_{b}=\varphi_{m}-{\left(\frac{kT}{4\pi R^{2}\gamma }\right)}^{1/3},$$

where, *φ_m_* is the volume fraction particles at maximum parking, *T* the temperature of the suspension, *R* the spherical radius for the particles, and *γ* the surface tension of the solvent.

In the present work, the ceramic slurry and the freezing process used were identical in all the cases, so the difference in porosity between the top and bottom parts should be caused only by the temperature of the ceramics slurry (*T*) during the freezing process. Although this temperature was not measured in this study, owing to the thermal conduction, the temperature of the ceramic slurry should decrease during the slurry solidification [10]. From Equation (1), we can therefore consider that the breakthrough concertation should increase with increasing the distance from the cold surface. For an equivalent initial slurry concentration, the fraction of micropores in the top part of the green body should be lower than that of the bottom part. Micropores were readily removed, facilitating the densification. As a result, the porosity of the top part was higher than that of the bottom part in a fired sample.

**Figure 6:**
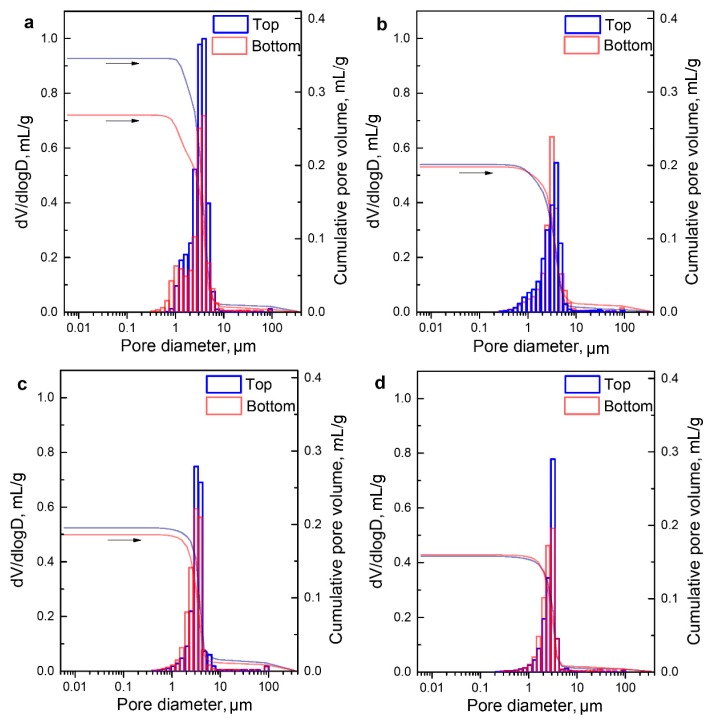
Pore size distribution and cumulative pore volume of the top and bottom parts of porous α-SiAlON samples fired at different conditions: (**a**): 1800 °C-1 h; (**b**): 1800 °C-2 h (**c**): 1900 °C-1 h and (**d**): 1900 °C-2 h.

Figure 6 gives pore size distribution and cumulative pore volume of the top and bottom parts of porous α-SiAlON samples fired under different conditions, revealing a unimodal pore size distribution in each case, with a peak at 3.1–4.0 μm. For a given solid loading, the pore size varied with firing condition and the location in a fired sample. With increasing the firing temperature from 1800 to 1900 °C and holding time from 1 to 2 h, the pore size decreased slightly, and the pores within the size range of 0.3–1.0 μm decreased significantly, further supporting that the micropores were readily removed during the densification process. Moreover, the top part of each fired sample exhibited a bigger overall pore size compared to the bottom part. This was consistent with the microstructural observation shown in Figure 3 and Figure 4.

### 3.3. Mechanical Properties

**Figure 7:**
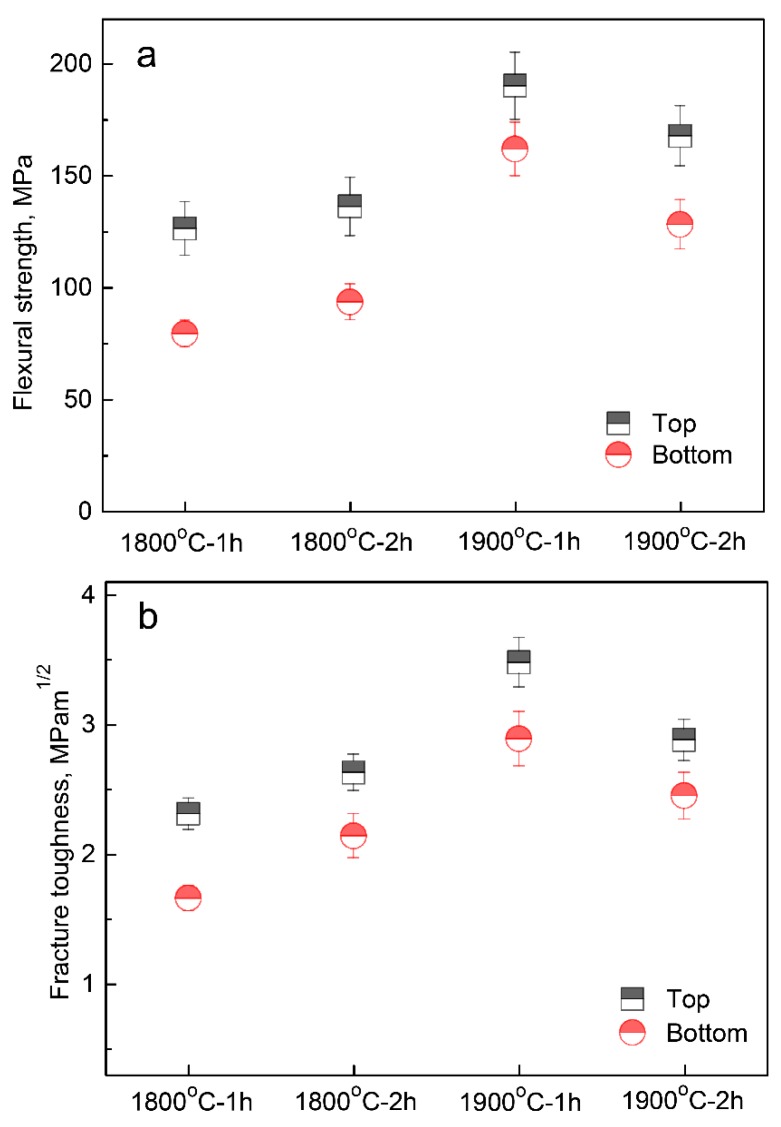
Flexural strength (**a**) and fracture toughness (**b**) of porous α-SiAlON samples fired at different conditions.

Figure 7 presents flexural strength and fracture toughness values of the top and bottom parts of fired porous α-SiAlON ceramics. With increasing the firing temperature from 1800 to 1900 °C, both flexural strength and fracture toughness increased, which can be attributed to the decrease in the porosity [13]. As mentioned earlier, the porosity decreased with increasing the firing temperature and time. However, for the samples fired at 1900 °C, the flexural strength and fracture toughness both slightly decreased upon extending the holding time to 2 h. This was probably due to grain coarsening and strong interfacial bonding.

Interestingly, for a given composition and initial slurry concentration, flexural strength and fracture toughness of the top part were both higher than those of the bottom part, even though the porosity of the former was higher than that of the latter (Figure 3), strongly suggesting that porosity was not the only factor that affected the mechanical properties. As already illustrated in Figure 4, the overall pore size of the bottom part was smaller and more intricate than that of the top part because of the decrease of solidification velocity with increasing the distance from the cold surface. Therefore, smaller and more intricate pores are believed to be responsible for the lower mechanical properties of the bottom part of as-prepared porous α-SiAlON ceramics. 

## 4. Conclusions

In this work, porous α-SiAlON ceramics were prepared by unidirectional freezing of camphene-based suspensions and subsequent firing at different conditions. Owing to the thermal resistance of the solidified layer, the solidification velocity decreased with increasing its thickness. Consequently, a typical gradient structure was formed in a fired sample, where pore size increased with increasing the distance from the bottom of the sample. Increase in the firing temperature and time led to decreased porosities in both top and bottom parts of fired samples. In addition, for an equivalent solid loading, the porosity of the top part was higher than that of the bottom part. Nevertheless, flexural strength and fracture toughness of the top part were both higher than those of the bottom part, suggesting that the porosity was not the only factor affecting the mechanical properties of as-prepared porous α-SiAlON ceramics. Therefore, it is necessary to carry out a systematic study in the nearest future to clarify the effects of pore morphology and size on mechanical properties of porous ceramics.
